# Digoxin Use to Control Ventricular Rate in Patients with Atrial Fibrillation and Heart Failure Is Not Associated with Increased Mortality

**DOI:** 10.1155/2015/314041

**Published:** 2015-12-14

**Authors:** Surbhi Chamaria, Anand M. Desai, Pratap C. Reddy, Brian Olshansky, Paari Dominic

**Affiliations:** ^1^Division of Cardiology and Center for Cardiovascular Diseases and Science, LSU Health Science Center, Shreveport, LA 71103, USA; ^2^Division of Cardiovascular Medicine, University of Iowa, Iowa City, IA 52242, USA

## Abstract

*Introduction.* Digoxin is used to control ventricular rate in atrial fibrillation (AF). There is conflicting evidence regarding safety of digoxin. We aimed to evaluate the risk of mortality with digoxin use in patients with AF using meta-analyses.* Methods.* PubMed was searched for studies comparing outcomes of patients with AF taking digoxin versus no digoxin, with or without heart failure (HF). Studies were excluded if they reported only a point estimate of mortality, duplicated patient populations, and/or did not report adjusted hazard ratios (HR). The primary endpoint was all-cause mortality. Adjusted HRs were combined using generic inverse variance and log hazard ratios. A multivariate metaregression model was used to explore heterogeneity in studies.* Results.* Twelve studies with 321,944 patients were included in the meta-analysis. In all AF patients, irrespective of heart failure status, digoxin is associated with increased all-cause mortality (HR [1.23], 95% confidence interval [CI] 1.16–1.31). However, digoxin is not associated with increased mortality in patients with AF and HF (HR [1.08], 95% CI 0.99–1.18). In AF patients without HF digoxin is associated with increased all-cause mortality (HR [1.38], 95% CI 1.12–1.71).* Conclusion.* In patients with AF and HF, digoxin use is not associated with an increased risk of all-cause mortality when used for rate control.

## 1. Introduction

Digoxin is the oldest cardiac medication currently being used in clinical practice. With its unique mechanism of action, digoxin has traditionally had a role in the management of heart failure and atrial fibrillation. Rigorous prospective trials evaluating digoxin did not exist until the 1980s. Initial clinical trials of digoxin comparing the drug to vasodilators, milrinone, and placebo and the subsequent digoxin withdrawal trials showed substantial evidence that digoxin offered symptomatic benefits to patients with heart failure, but mortality benefits of digoxin remain controversial. The Digitalis Investigator Group (DIG) trial sponsored by the NIH, designed to detect mortality differences with digoxin use in patients with sinus rhythm and systolic dysfunction, failed to show any survival benefit with digoxin use [1]. A post hoc analysis of the study more than a decade later showed that patients who had higher serum digoxin concentrations had an absolute 11.8% increase in all-cause mortality. While recent retrospective and prospective studies show an association of digoxin use with increased mortality in patients with heart failure who are in sinus rhythm, a Cochrane meta-analysis of 13 studies showed a neutral effect on mortality [2]. Recent meta-analysis has shown an association of increased mortality with use of digoxin as a rate-controlling agent in patients with atrial fibrillation only [3, 4]. The safety and benefit of digoxin in patients with atrial fibrillation and heart failure for rate control continue to be controversial. Here we used meta-analytical techniques to assess the risk of mortality with digoxin use in patients with atrial fibrillation and heart failure.

## 2. Methods

Our analysis is based on the guidelines of the meta-analysis of observational studies in the Epidemiology Group [5].

### 2.1. Inclusion and Exclusion Criteria

We included prospective or retrospective observational studies with a primary objective to analyze the association between digoxin and all-cause mortality in patients with atrial fibrillation with or without heart failure. Titles and abstracts were evaluated and rejected after initial screening according to the following inclusion and exclusion criteria: studies were included if (1) digoxin was compared to no digoxin or any other rate-controlling drug in patients with atrial fibrillation; (2) the duration of follow-up was at least 6 months; (3) adjusted hazard ratio was reported; (4) all-cause mortality was the endpoint.

Studies were excluded if (1) they included only patients with postoperative atrial fibrillation; (2) there was no control group; (3) they included only patients with heart failure; (4) adjusted hazard ratios were not reported. Abstracts alone were not considered.

### 2.2. Search Strategies

We searched MEDLINE (1966–2015) and Web of Science (1966–2015) databases to identify relevant studies. We used the following keywords: “digoxin,” “atrial fibrillation,” “heart failure,” and “mortality.” In addition, the “Related Articles” feature on PubMed was used and a manual search was conducted using bibliographies of included studies and review articles on this topic. Titles and abstracts were reviewed independently by two reviewers (Surbhi Chamaria and Anand M. Desai). Differences were resolved by consensus.

### 2.3. Quality Assessment and Data Extraction

The quality of each study was evaluated according to the guidelines developed by the United States Preventive Task Force and the Evidence-Based Medicine Working Group [6, 7] The following characteristics were assessed: (1) inclusion and exclusion criteria; (2) representative study sample; (3) explanation of sample selection; (4) full specification of clinical and demographic variables; (5) follow-up at least 6 months; (6) reported loss of follow-up; (7) definition of outcomes and outcome assessment; and (8) adjustment of possible confounders in multivariate analyses. Studies were graded as poor if they met <3 criteria, fair if they met 3–5 criteria, and good if they met ≥5 criteria.

Two reviewers (Surbhi Chamaria and Anand M. Desai) extracted (1) publication details including first author's last name and year of publication; (2) study design; (3) characteristics of the study population including: gender, race, age, and comorbidities (hypertension, diabetes, previous strokes, ejection fraction, and chronic kidney disease); (4) variables included in the multivariate analyses; and (5) adjusted hazard ratio (HR) with 95% confidence interval (CI) from the multivariate analyses. All studies used a cox proportional hazards analysis to calculate adjusted HR. Wherever the studies used a propensity score matching, HRs for this meta-analysis were extracted from the propensity matched analysis.

### 2.4. Statistical Analysis

The degree of association between digoxin and all-cause mortality in patients with atrial fibrillation, with and without heart failure, was measured as a HR. All the studies employed Cox proportional hazard models to examine association of digoxin and mortality, thereby enabling the use of one consistent measure throughout. One study was excluded as it reported relative risk and no HR [8]. Risk estimates (HRs) were extracted. These studies reported use of multivariate and propensity score models to adjust for potential confounders including age, sex, heart failure, hypertension, chronic kidney disease, beta-blocker use, aspirin use, warfarin use, and history of previous stroke.

A prespecified subgroup analysis was performed based on whether heart failure population was included and reported in the study. HRs were transformed logarithmically as they did not follow a normal distribution. The standard error was calculated from Log HR and the corresponding 95% confidence interval (CI). The inverse variance method was used to achieve a weighted estimate of the combined overall effect. Results for heterogeneity were examined by the forest plots and calculating a *Q* statistic, which we compared with the *I* ^2^ index [9]. Significant heterogeneity was considered present at the 5% level of significance (for the *Q* test) and values of *I* ^2^ exceeding 56% [9]. Overall analyses (*Q* test *P* < 0.01; *I* ^2^ = 85%) and all subgroups except patients with atrial fibrillation and heart failure only exhibited significant heterogeneity. This prompted us to adopt the random effect model. All primary analyses were performed using Cochrane's review manager 5.2. This model allowed a distribution of the true effect size rather than assuming one true effect size. It took into account within-study and between-study variance.

The underlying heterogeneity further prompted us to perform metaregression analysis to investigate factors contributing to heterogeneity and if our study outcome (all-cause mortality) was affected by factors other than our primary treatment (digoxin) [10, 11]. We adopted a weighted regression random effect model and carried out a multivariant regression using three predetermined factors including hypertension, left ventricular ejection fraction, and prior history of stroke using comprehensive meta-analysis version 3. These factors were selected based on factors shown to increase mortality with digoxin in individual studies and on availability of data for majority of the studies included. A two-sided *P* value < 0.05 was regarded as significant for all analyses. Data was represented as forest plots for primary analysis. Potential publication bias was assessed with the Egger test and represented graphically with Begg's funnel plots of the natural log of the HR versus its standard error [12].

## 3. Results

**Figure 1:**
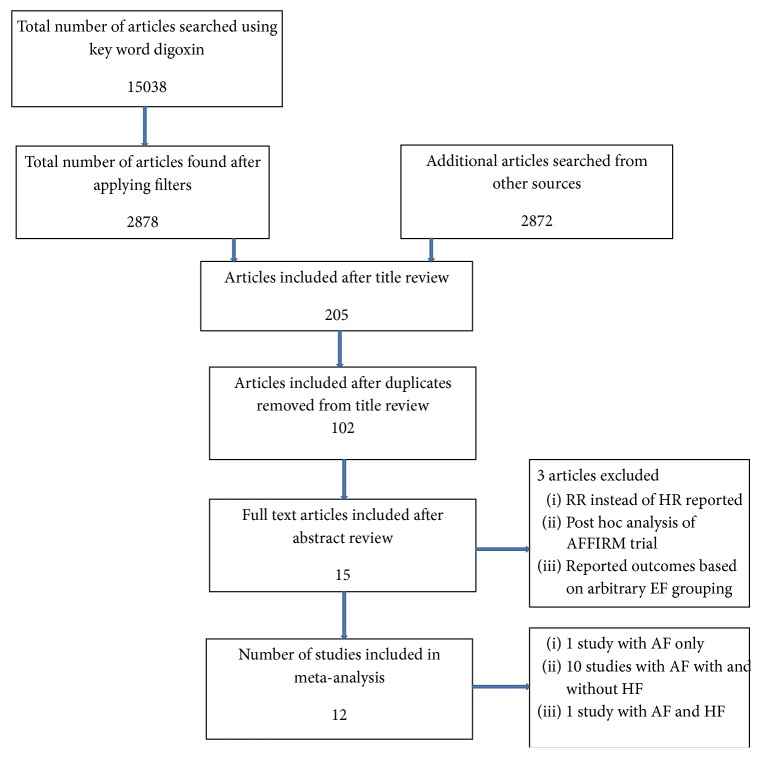
Prisma flow diagram for study selection.

The literature search yielded 17910 potential studies—15038 by key words search and 2872 from other sources (Figure 1). After screening titles and abstracts and removing duplicated studies, 17808 articles were excluded. An additional 87 articles were excluded because they were either review articles or did not satisfy our inclusion criteria. Out of the 15 articles selected for detailed evaluation three were excluded from analysis for one or more of the following reasons: (1) not reporting HR for mortality; (2) duplicating patient population from another study; (3) excluding 22% of patients from the AFFIRM trial as data regarding their previous use of digoxin prior to the trial was missing; and (4) reporting outcomes based on left ventricular ejection fraction less than or more than 30%, an arbitrary delineation that differed from the rest of the studies and did not form part of our prespecified analysis. All the studies included in the meta-analyses used digoxin primarily for rate control of atrial fibrillation and not for the management of heart failure.

### 3.1. Patient Population and General Characteristics of Included Studies

For our primary comparison evaluating effect of digoxin on patients with atrial fibrillation, we included 12 studies with 321,944 patients. Ten of these 12 studies included atrial fibrillation patients both with and without heart failure, but only three of these ten studies reported separate outcomes for patients with and without heart failure and seven did not. Of the remaining two studies, one included only patients with atrial fibrillation without heart failure and one included only patients with atrial fibrillation and heart failure.

Baseline characteristics of the included studies for our primary comparison are shown in Table 1. The baseline characteristics of the patients included in these trials based on the treatment with digoxin are presented in Table 2. The 12 studies varied in size, characteristics of patient populations, ancillary therapy for heart failure, and use of antiplatelet or anticoagulation drugs for stroke prevention.

Importantly, for all studies, treatment with digoxin was not randomized. Majority of the studies did not state the dose of digoxin used and only one study [13] measured the level of digoxin during the follow-up period. Seven out of the twelve studies commented on the number of patients with chronic kidney disease, out of which only one study [14] reported that the use of digoxin in patients with chronic kidney disease correlated significantly with increased mortality.

### 3.2. Results of Component Studies

In all the four studies that considered the effect of digoxin in patients with AF and no HF there was an increase in all-cause mortality [13, 15–17]. In all the four studies, patients were older and had more baseline comorbidities compared to other studies. In the study by Freeman et al. [13], mean serum digoxin concentration was higher among patients who died in the digoxin group.

**Figure 2:**
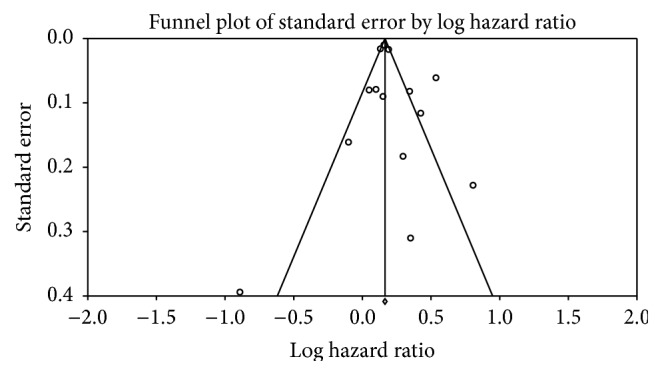
Funnel plot.

Of the seven studies that considered all-cause mortality in patients with atrial fibrillation irrespective of heart failure status, four of them showed an increase in all-cause mortality with the use of digoxin [18–21]. In all of these studies digoxin users were older and had more baseline co-morbidities as compared with non-digoxin users. Three studies in this group did not show an increase in mortality [22–24]. Of the four studies that considered patients with atrial fibrillation and concomitant heart failure, three studies showed that digoxin had no effect on all cause mortality [14, 15]. Analysis of the Begg's funnel plot of the included studies showed no significant publication bias (Figure 2).

### 3.3. Results of Meta-Analysis and Metaregression

**Figure 3:**
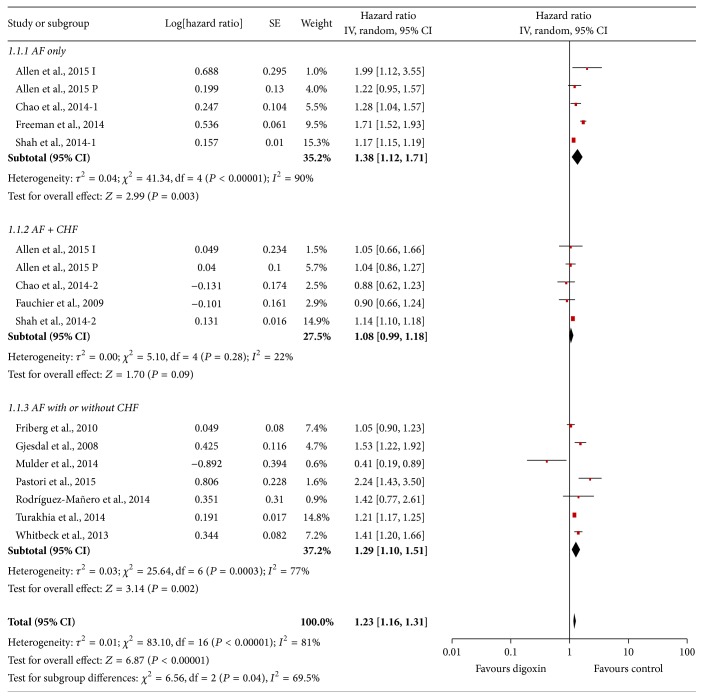
Forest plot showing combined effect of digoxin on all-cause mortality in studies with patients with atrial fibrillation only, atrial fibrillation with heart failure only, and atrial fibrillation with or without heart failure.

Results of the combined analysis of adjusted HR for all-cause mortality for all patients with atrial fibrillation irrespective of heart failure status showed that patients prescribed digoxin had almost a 25% higher risk of mortality compared to those not on digoxin (HR 1.23, 95% CI 1.16–1.31, Figure 3). However, a prespecified subgroup analysis performed for the purpose of this study showed that in patients with atrial fibrillation and heart failure, there was no increase in all-cause mortality with digoxin use (HR 1.08, 95% CI 0.99–1.18). All-cause mortality was higher with the use of digoxin in patients with atrial fibrillation alone (HR 1.38, 95% CI 1.12–1.71).

**Figure 4:**
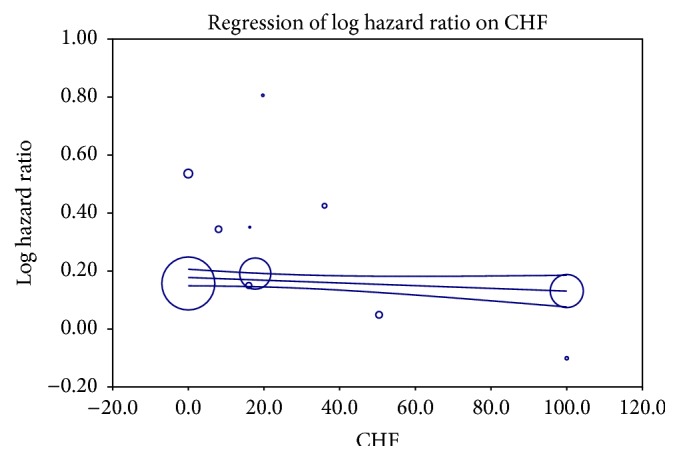
Effect of percentage of patients with CHF in individual studies on mortality risk of digoxin using a multivariate metaregression model; increased percentage of patients with CHF on the *x*-axis correlates with decreased HR on the *y*-axis.

**Figure 5:**
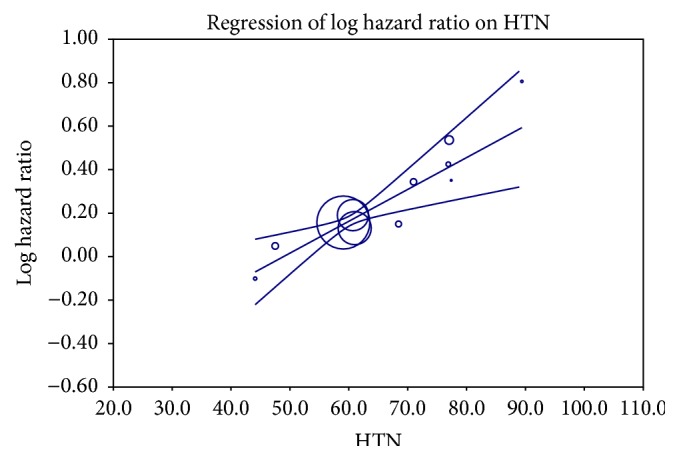
Effect of percentage of hypertensive patients in individual studies on mortality risk of digoxin using a multivariate metaregression model including hypertension, heart failure, and previous stroke; increased percentage of patients with hypertension on the *x*-axis correlated with increased HR on the *y*-axis.

We attempted to explore the reasons behind the heterogeneity among the included studies and to investigate factors influencing the effect of digoxin on mortality, by performing a metaregression analysis. Confirming our findings of the subgroup analysis of studies with atrial fibrillation and heart failure, a univariate metaregression analysis showed that the percentage of patients with heart failure in the included studies negatively correlated with the hazard ratio for all-cause mortality (*P* = 0.04). In addition, the number of patients with hypertension and history of previous stroke positively correlated with increased mortality. A multivariant metaregression model including all the three factors, hypertension, heart failure, and history of previous stroke, showed that heart failure (*P* = 0.03, Figure 4) and hypertension (*P* < 0.001, Figure 5) but not previous history of stroke (*P* = 0.26) strongly correlated with increased mortality in the studies and they contributed almost entirely to the heterogeneity between studies (*R* ^2^ analog = 1). Of note, mean age and percentage of beta-blocker use in the study population did not have any correlation with the hazard ratios when used as covariates in the metaregression.

## 4. Discussion

Although digoxin is widely used as a rate-controlling drug in atrial fibrillation, there is a paucity of randomized controlled trials evaluating its safety. The long-term effect of digoxin on mortality and heart failure hospitalization in HF patients was studied in the prospective randomized trial Digitalis Investigators Group (DIG). The study showed that digoxin compared with placebo had no effect on survival when used with angiotensin converting enzyme inhibitors and diuretics. A meta-analysis of 13 studies of digoxin in heart failure confirmed that digoxin had no effect on mortality in heart failure but all the studies in the analysis excluded patients with atrial fibrillation. Additionally, the results of the meta-analysis heavily relied on the DIG trial. Our meta-analysis showed a similar outcome in atrial fibrillation patients with heart failure, that is, no effect on mortality. In patients with atrial fibrillation alone without coexisting heart failure, the combined hazard ratio showed an increased risk of death.

Two recent meta-analyses assessing the effect of digoxin in patients with atrial fibrillation were published. The study by Ouyang et al. [3] concluded that digoxin increases mortality in patients with atrial fibrillation and heart failure. This difference in results stems from key disparities in inclusion criteria and data extraction between the two studies: (1) HR of a subgroup analysis reported by Mulder et al. for AF patients with NT pro-BNP more than 1003 pg/mL was taken as a surrogate for heart failure by Ouyang et al. while we did not make such assumptions. (2) While our study did not include separate outcomes for AF patients with and without heart failure for Rodríguez-Mañero et al. and Whitbeck et al., two studies that self-reported missing data on heart failure and LV systolic dysfunction data in a substantial number of their patients, Ouyang et al. proceeded to include that data in their subgroup analysis. (3) Ouyang et al. included risk estimates from Whitbeck et al. [18] and Gheorghiade et al. [25], two studies that performed post hoc analyses on the AFFIRM trial, thus duplicating the population. We excluded the report by Gheorghiade to avoid duplication and also because the analysis excluded 22% of the AFFIRM study population due to missing data on digoxin use prior to the start of the trial. (4) Further, Ouyang et al. have included outcome estimates from Georgiopoulou et al. [26], a study that reported only a combined end point of time to death or urgent transplantation or left ventricular assist device implantation. We excluded this study from our analysis as the study by Georgiopoulou et al. was primarily a study of the effect of digoxin in patients with heart failure and they did not report a separate hazard ratio for mortality.

The second meta-analysis by Vamos et al. [4] reported increased mortality in patients with HF on digoxin but a subgroup analysis of studies with HF and AF noted no difference in mortality between the groups, similar to our study. Our analysis is updated with the recently published results from a large AF cohort that was not included in Vamos et al. Furthermore, multiple studies categorized as digoxin use in patients with atrial fibrillation without heart failure by Vamos et al. included up to 50% patients with heart failure (Table 1) making their conclusions unclear. Moreover, the relative risks from the study by Hallberg et al. [8] have been combined with the adjusted HRs from other studies by both Ouyang et al. and Vamos et al., while we excluded Hallberg et al. to avoid combining a cumulative risk measure with an instantaneous risk measure. Finally, our analysis also includes a metaregression showing that the percentage of HF patients reported in all the studies negatively correlated with the hazard ratio for all-cause mortality, corroborating the overall evidence that HF might blunt the effect of digoxin on the all-cause mortality of patients with AF.

The result of our meta-analysis is clinically very relevant. In patients with HF, pharmacologic therapy for rate control in AF is limited due to negative inotropic effects of commonly used drugs that prolong refractoriness of the AV node including beta-blockers and nondihydropyridine calcium channel blockers. In addition, these drugs also cause hypotension in HF patients with severe systolic dysfunction. In this context, digoxin is an essential alternate for rate control and continues to be recommended for patients with HF (Class IC) in the recent guidelines for the management of patients with AF [27]. Therefore, any association of mortality with digoxin use in this patient population has to be proven beyond doubt. A post hoc analysis of the DIG trial showed that the effectiveness of digoxin in patients with heart failure depended on serum digoxin concentration (SDC). Only one component study in the recent meta-analyses including ours reported SDC [13] and none correlated the outcomes with SDC, a potential confounding factor.

The reasons behind the difference in the effect of digoxin on mortality in patients with atrial fibrillation and heart failure and patients with atrial fibrillation alone are unclear. It has been shown that the beneficial effects of digoxin in heart failure in patients with sinus rhythm are due to its neurohormonal modulation effect and inotropic effect which vary with serum digoxin concentration. At low doses, neurohormonal effects provide symptomatic relief with a positive inotropic effect, but at increasing doses, the inotropic effect may increase myocardial oxygen consumption and arrhythmogenicity. In contrast, in patients without heart failure, beneficial effects may be overshadowed by the potential harmful effects of digoxin [28].

Digoxin toxicity can cause every known disturbance of cardiac impulse formation and propagation leading to significant arrhythmias. The positive inotropic action of digoxin is likely due to increased intracellular calcium. This increased intracellular calcium load not only augments contractility, but also can initiate delayed after depolarization and triggered arrhythmias [29]. Digoxin can initiate ectopic activity and produce bradycardia including AV block [30], plausible mechanisms of increased mortality observed with digoxin use in atrial fibrillation in the absence of heart failure.

*Limitations*. The present meta-analysis is based on retrospective and prospective nonrandomized trials and consequently has limitations in the wider application of the results of our analysis. Specifically, patients who are prescribed digoxin in nonrandomized retrospective studies may be inherently different from patients not prescribed digoxin and this is clear from the comorbidities seen in digoxin patients. While the Cox proportional hazards model (all studies) and propensity score models (all but 3 studies) used by individual studies included in our analysis somewhat mitigate this weakness by adjusting for known variables, they do not completely eliminate it. Also, our meta-analysis includes some large registries and cohort studies that could potentially influence the effect sizes but by employing a random effect model, we expect to moderate such impact. Warfarin use in component studies ranged from 33% to 100% and was not reported in some, thus limiting our ability to analyze and understand the effect of warfarin use as a covariate. Finally, guideline directed medical therapy use specifically in patients with CHF within the study cohort in component studies was not reported. In the two studies with 100% CHF population, a large proportion of patients was not on ACE inhibitors and beta-blockers, limiting the interpretation of our results.

## 5. Conclusion

This meta-analysis of nonrandomized studies shows that digoxin is not associated with increased all-cause mortality when used as a rate-controlling drug in patients with atrial fibrillation with coexistent heart failure but it is associated with increased mortality when used in patients with atrial fibrillation alone. Large, well-designed, randomized controlled trials are needed to further address this issue.

## Figures and Tables

**Table 1:** Study characteristics evaluating the association of digoxin and the risk of mortality in patients with atrial fibrillation.

First author	Study design	Digoxin group	Control	Age (yrs)	Male (%)	CHF (%)	Follow-up (yrs)	Primary endpoints	Analysis method	Study quality
Chao (2014) [15]	Prospective	829	3,952	70.8 ± 12.5	52.1	23.8	4.3	All-cause mortality	CRM, NPM	Good
Fauchier (2009) [14]	Prospective	402	867	76 ± 13	56.2	100.0	2.4	All-cause mortality	CPHM, NPM	Good
Shah^*∗*^ (2014) [16]	Retrospective	23,200	77,399	79.4 ± 7.2	42.8	NA	4.2	All-cause mortality	CPHM, PM	Good
Shah^*∗∗*^ (2014) [16]		15,181	24,331	80.1 ± 7.4	50.0	100.0	3.1	All-cause mortality	CPHM, PM	Good
Friberg (2010) [22]	Prospective	802	2,022	78	45.7	63.7	4.6	All-cause mortality	CRM, PM	Good
Whitbeck (2013) [18]	Retrospective	2,153	1,905	NA	NA	12.0	3.5	All-cause mortality	CPHM, PM	Good
Gjesdal (2008) [19]	Retrospective	3,911	3,418	71 ± 9	66.9	45.3	0.8	All-cause mortality	CPHM, NPM	Good
Turakhia (2014) [20]	Prospective	28,679	93,786	71.7 ± 10.2	98.5	21.3	2.8	All-cause mortality	CPHM, PM	Good
Mulder (2014) [24]	Retrospective	284	324	68 ± 8	59.9	8.5	3	All-cause mortality	CPHM, NPM	Good
Rodríguez-Mañero (2014) [23]	Retrospective	212	565	76.9 ± 8.4	46.2	19.8	2.9	All-cause mortality, survival free of admission	CPHM, NPM	Good
Freeman (2014) [13]	Retrospective	4,858	22,430	71.9 ± 11.9	50.2	0.0	1.2	All-cause mortality	CPHM, PM	Good
Pastori (2015) [21]	Prospective	171	644	74.4 ± 7.2	53.2	25.7	2.7	All-cause mortality	CPHM, PM	Good
Allen (2015) [17]	Prospective	2948	6671	76	55.7	21.9	1.8	All-cause mortality	CFM, PM	Good

CHF: congestive heart failure; NA: not available; CRM: Cox regression model; CPHM: Cox proportional hazards model; PM: propensity matching; NPM: not propensity matched; CFM: Cox frailty model.

Shah^*∗*^: study characteristics who only had atrial fibrillation.

Shah^*∗∗*^: study characteristics in patients who had both atrial fibrillation and congestive heart failure.

**Table 2:** Baseline characteristics of patients included in the component studies.

First author	Sex (%)	Age (yrs)	CKD (%)	BB (%)	ACEi (%)	CAD (%)	DM (%)	CHF (%)	HTN (%)	Stroke (%)	ASA (%)	Coumadin (%)
Chao (2014) [15]	53.4	68.0	8.1	16.2	18.5		32.1	16.0	68.5	19.8		
Fauchier (2009) [14]	60.0	74.3	9.3	50.0	77.1	21.4	17.0	100.0	44.1	4.7	31.2	58.0
Shah^*∗*^ (2014) [16]	45.6	79.0	12.5	41.8	29.0	37.8	22.4	0.0	59.1	7.8	29.9	60.3
Shah^*∗∗*^ (2014) [16]	50.0	80.2	28.3	50.3	47.4	58.9	31.4	100.0	61.0	4.6	36.8	63.0
Friberg (2010) [22]	52.0	75.5	2.0	51.0	36.0	20.0	19.0	50.4	47.5	17.5	40.5	44.5
Whitbeck (2013) [18]				30.0		38.0		8.0	71.0	13.5		
Gjesdal (2008) [19]	69.0	71.0		5.0		44.8	23.4	36.0	76.9	21.1	16.3	
Turakhia (2014) [20]	98.4	72.0	36.0	60.1	55.2		28.5	17.7	60.7	5.9	15.7	58.6
Mulder (2014) [24]	65.2	68.0		66.4	51.2	17.9	11.2	6.9	60.9			
Rodríguez-Mañero (2014) [23]	52.4	75.7	9.8	31.1		17.8	28.0	16.3	77.4	4.3		
Freeman (2014) [13]	53.5	71.2	35.5	56.1	35.2	5.3	23.5	0.0	77.1	35.8	6.8	33.1
Pastori (2015) [21]	55.9	73.5		40.8	69.5	48.2	21.4	19.7	89.4	16.3	8.1	100.0
Allen P (2015) [17]	56.9	75.5				31.7	31.5	13.0				
Allen I (2015) [17]	57.2	75.5				31.7	31.5	10.0				

CKD: chronic kidney disease, BB: beta-blocker, ACEi: ace inhibitor, CAD: coronary artery disease, DM: diabetes mellitus, CHF: congestive heart failure, HTN: hypertension, and ASA: aspirin.

Shah^*∗*^: metaregression done on patients who only had atrial fibrillation.

Shah^*∗∗*^: metaregression done on patients who had both atrial fibrillation and congestive heart failure

Allen P: prevalent digoxin group; Allen I: incident digoxin group.

## References

[1] Digitalis Investigation Group (1997). The effect of digoxin on mortality and morbidity in patients with heart failure. *The New England Journal of Medicine*.

[2] Hood W. B., Dans A. L., Guyatt G. H., Jaeschke R., McMurray J. J. (2004). Digitalis for treatment of congestive heart failure in patients in sinus rhythm. *Cochrane Database of Systematic Reviews*.

[3] Ouyang A.-J., Lv Y.-N., Zhong H.-L. (2015). Meta-analysis of *digoxin* use and risk of mortality in patients with atrial fibrillation. *The American Journal of Cardiology*.

[4] Vamos M., Erath J. W., Hohnloser S. H. (2015). Digoxin-associated mortality: a systematic review and meta-analysis of the literature. *European Heart Journal*.

[5] Stroup D. F., Berlin J. A., Morton S. C. (2000). Meta-analysis of observational studies in epidemiology: a proposal for reporting. *The Journal of the American Medical Association*.

[6] Harris R. P., Helfand M., Woolf S. H. (2001). Current methods of the US Preventive Services Task Force: a review of the process. *American Journal of Preventive Medicine*.

[7] Levine M., Walter S., Lee H., Haines T., Holbrook A., Moyer V. (1994). Users' guides to the medical literature. IV. How to use an article about harm. Evidence-Based Medicine Working Group. *The Journal of the American Medical Association*.

[8] Hallberg P., Lindbäck J., Lindahl B., Stenestrand U., Melhus H. (2007). Digoxin and mortality in atrial fibrillation: a prospective cohort study. *European Journal of Clinical Pharmacology*.

[9] Higgins J. P. T., Thompson S. G. (2002). Quantifying heterogeneity in a meta-analysis. *Statistics in Medicine*.

[10] Thompson S. G., Higgins J. P. T. (2002). How should meta-regression analyses be undertaken and interpreted?. *Statistics in Medicine*.

[11] Thompson S. G., Sharp S. J. (1999). Explaining heterogeneity in meta-analysis: a comparison of methods. *Statistics in Medicine*.

[12] Egger M., Smith G. D., Schneider M., Minder C. (1997). Bias in meta-analysis detected by a simple, graphical test. *British Medical Journal*.

[13] Freeman J. V., Reynolds K., Fang M. (2015). Digoxin and risk of death in adults with atrial fibrillation: the ATRIA-CVRN study. *Circulation: Arrhythmia and Electrophysiology*.

[14] Fauchier L., Grimard C., Pierre B. (2009). Comparison of beta blocker and digoxin alone and in combination for management of patients with atrial fibrillation and heart failure. *The American Journal of Cardiology*.

[15] Chao T.-F., Liu C.-J., Chen S.-J. (2014). Does digoxin increase the risk of ischemic stroke and mortality in atrial fibrillation? A nationwide population-based cohort study. *Canadian Journal of Cardiology*.

[16] Shah M., Avgil Tsadok M., Jackevicius C. A., Essebag V., Behlouli H., Pilote L. (2014). Relation of digoxin use in atrial fibrillation and the risk of all-cause mortality in patients ≥65 years of age with versus without heart failure. *The American Journal of Cardiology*.

[17] Allen L. A., Fonarow G. C., Simon D. N. (2015). Digoxin use and subsequent outcomes among patients in a contemporary atrial fibrillation cohort. *Journal of the American College of Cardiology*.

[18] Whitbeck M. G., Charnigo R. J., Khairy P. (2013). Increased mortality among patients taking digoxin—analysis from the AFFIRM study. *European Heart Journal*.

[19] Gjesdal K., Feyzi J., Olsson S. B. (2008). Digitalis: a dangerous drug in atrial fibrillation? An analysis of the SPORTIF III and V data. *Heart*.

[20] Turakhia M. P., Santangeli P., Winkelmayer W. C. (2014). Increased mortality associated with digoxin in contemporary patients with atrial fibrillation findings from the TREAT-AF study. *Journal of the American College of Cardiology*.

[21] Pastori D., Farcomeni A., Bucci T. (2015). Digoxin treatment is associated with increased total and cardiovascular mortality in anticoagulated patients with atrial fibrillation. *International Journal of Cardiology*.

[22] Friberg L., Hammar N., Rosenqvist M. (2010). Digoxin in atrial fibrillation: report from the Stockholm Cohort study of Atrial Fibrillation (SCAF). *Heart*.

[23] Rodríguez-Mañero M., Otero-Raviña F., García-Seara J. (2014). Outcomes of a contemporary sample of patients with atrial fibrillation taking Digoxin: results from the AFBAR study. *Revista Espanola de Cardiologia*.

[24] Mulder B. A., Van Veldhuisen D. J., Crijns H. J. G. M. (2014). Digoxin in patients with permanent atrial fibrillation: data from the RACE II study. *Heart Rhythm*.

[25] Gheorghiade M., Fonarow G. C., van Veldhuisen D. J. (2013). Lack of evidence of increased mortality among patients with atrial fibrillation taking digoxin: findings from post hoc propensity-matched analysis of the AFFIRM trial. *European Heart Journal*.

[26] Georgiopoulou V. V., Kalogeropoulos A. P., Giamouzis G. (2009). Digoxin therapy does not improve outcomes in patients with advanced heart failure on contemporary medical therapy. *Circulation—Heart Failure*.

[27] January C. T., Wann L. S., Alpert J. S. (2014). 2014 AHA/ACC/HRS guideline for the management of patients with atrial fibrillation: a report of the American College of Cardiology/American Heart Association Task Force on Practice Guidelines and the Heart Rhythm Society. *Journal of the American College of Cardiology*.

[28] Rathore S. S., Curtis J. P., Wang Y. F., Bristow M. R., Krumholz H. M. (2003). Association of serum digoxin concentration and outcomes in patients with heart failure. *The Journal of the American Medical Association*.

[29] Mazur A., Anderson M. E. (1998). Digoxin and cardiac arrhythmias: an update. *Cardiac Electrophysiology Review*.

[30] Kastor J. A., Yurchak P. M. (1967). Recognition of digitalis intoxication in the presence of atrial fibrillation. *Annals of Internal Medicine*.

